# Transformer-Based Multiple-Object Tracking via Anchor-Based-Query and Template Matching

**DOI:** 10.3390/s24010229

**Published:** 2023-12-30

**Authors:** Qinyu Wang, Chenxu Lu, Long Gao, Gang He

**Affiliations:** State Key Laboratory of Integrated Service Networks, School of Telecommunications Engineering, Xidian University, No. 2, South Taibai Street, Hi-Tech Development Zone, Xi’an 710071, China; lcx031478@gmail.com (Q.W.); 22011210922@stu.xidian.edu.cn (C.L.); ghe@xidian.edu.cn (G.H.)

**Keywords:** video processing, multiple-object tracking, anchor-based query, transformer, template matching

## Abstract

Multiple object tracking (MOT) plays an important role in intelligent video-processing tasks, which aims to detect and track all moving objects in a scene. Joint-detection-and-tracking (JDT) methods are thriving in MOT tasks, because they accomplish the detection and data association in a single stage. However, the slow training convergence and insufficient data association limit the performance of JDT methods. In this paper, the anchor-based query (ABQ) is proposed to improve the design of the JDT methods for faster training convergence. By augmenting the coordinates of the anchor boxes into the learnable queries of the decoder, the ABQ introduces explicit prior spatial knowledge into the queries to focus the query-to-feature learning of the JDT methods on the local region, which leads to faster training speed and better performance. Moreover, a new template matching (TM) module is designed for the JDT methods, which enables the JDT methods to associate the detection results and trajectories with historical features. Finally, a new transformer-based MOT method, ABQ-Track, is proposed. Extensive experiments verify the effectiveness of the two modules, and the ABQ-Track surpasses the performance of the baseline JDT methods, TransTrack. Specifically, the ABQ-Track only needs to train for 50 epochs to achieve convergence, while that for TransTrack is 150 epochs.

## 1. Introduction

Multiple object tracking (MOT) is a thriving field in intelligent video processing, and has wide applications in autonomous vehicles, video surveillance, and intelligent transportation [1]. The objective of the MOT methods is to estimate the movement states of objects and maintain identifications of those objects within a single video stream. Despite the numerous efforts dedicated to MOT, the design of efficient and robust trackers remains a challenge, primarily due to the need to address two critical sub-tasks within a single tracker: detection and data association [2].

**Figure 1:**
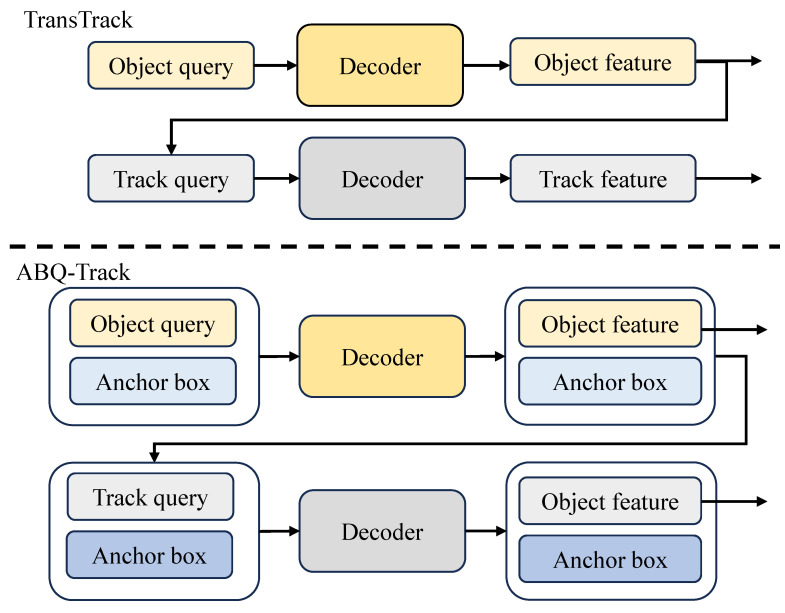
Comparison of the pipeline of the classical transformer-based MOT tracker, TransTrack, and the proposed tracker, ABQ-Track.

In the field of Multiple Object Tracking (MOT), Tracking-by-Detection (TBD) has been the domain framework, which utilized detection methods to find objects in the scenes, and applied the data association to join the detection results into the trajectories. The detection and data association were conducted separately, which neglected the inner link between the two procedures [3]. Furthermore, the training of the TBD methods was complicated since the training procedures of models for detection and data association were different [4,5]. To overcome these problems, Joint-Detection-and-Tracking (JDT) methods were proposed, which performed the detection and data association in one stage [6,7,8]. Specifically, the JDT modified the transformer-based network for MOT, which consists of the encoder and the decoder. The encoder is utilized for feature enhancement, and the decoder is implemented for detecting the objects based on the object queries and the tracker queries, respectively. As shown in Figure 1, the decoder detects the objects in the images by applying the object queries, which are learned in the training procedure. Meanwhile, the queries obtained in the previous image (track queries) were fed into the decoder, which shared the same structure with the decoder for detection, to estimate the location of the object in different trajectories. By computing the IoU scores of the detection results based on the two set of queries, the identifications were maintained. In general, the JDT methods unified the detection and data association in one network, and competitive performance was achieved.

However, the JDT methods face challenges related to extended training times due to inappropriate design of the learnable queries [9]. In the cross-attention module of the decoder, an attention map is learned with the queries and keys. The two queries, the object queries and the track queries, are applied to match the content and spatial information in the keys [10], and the spatial information in the queries is not encoded with the same pattern [9]. Consequently, more training epochs are needed for the JDT methods to learn strong enough queries. Additionally, JDT associates detection results with tracking results from previous frames with spatial information, the IoU scores, which is insufficient for maintaining stable trajectories. Objects within a trajectory may become occluded by other objects in the background, and their appearance may undergo significant changes [11,12]. The data association methods in JDT lack an explicit model for maintaining stable trajectories.

To address the above issues, a new MOT method, ABQ-Track, is proposed. To begin with, the explicit prior spatial knowledge for each trajectory is introduced by 4D anchor box (ABQ) and added into each query corresponding to the trajectory. The 4D anchor boxes consist of the locations and sizes of objects in the previous image, helping the data association module focus on a local region corresponding to the object in each trajectory, and the locations and sizes are encoded with the same ways as the positional embedding for the keys. Consequently, the tracker augmented with additional spatial prior knowledge achieves better performance, while the network is trained for fewer epochs. Additionally, the template matching (TM) is implemented to stabilize the trajectories. In addition to associating the trajectories and the detection results with spatial information, the TM enables the JDT to accomplish the data association with appearance information. In previous images, the TM reserves the appearances of the objects in different trajectories and fuses these appearances to obtain templates, which represent the appearances of objects in different trajectories. By comparing the templates with the features corresponding to the detection results, the ABQ-Track associates the data with appearance information. Finally, the ABQ and TM are instanced in the classical JDT method, TransTrack [7], as shown in Figure 1. Extensive experiments verify the effectiveness of the proposed methods. ABQ-Track achieves better performance than TransTrack on MOT-17 [13] and MOT-20 [14], and only needs to be trained for 50 epochs, which is much faster than that of TransTrack.

In general, the main contributions of this work can be summarized as follows:

(1) The ABQ approach is proposed to reduce the convergence time and improve the discriminative ability of the JDT method by incorporating explicit spatial knowledge into the queries.
(2) The TM method is introduced to stabilize the trajectories by associating the trajectories and detection results with historical appearance information in the trajectories.
(3) A new JDT method based on the two methods, ABQ-Track, is proposed. Extensive experiments are conducted, and the results verify the effectiveness of the proposed methods. Moreover, the ABQ-Track surpasses the performance of the other JDT methods, TransTrack, in faster training convergence.

## 2. Related Work

With the development of intelligent sensors, intelligent video-processing methods have been thriving for decades. Multi-object tracking (MOT) is an important task in intelligent video processing owing to its wide applications in the real-world. MOT focuses on tracking an unknown number of objects within given categories [15]. In this section, the two main pipelines, tracking-by-detection (TBD) and joint-detection-and-tracking (JDT), are reviewed firstly. The transformer architecture is then introduced since the method proposed in this paper is based on it.

### 2.1. Tracking-by-Detection

TBD methods directly applied the given detection results in the MOT datasets or the detection results outputted by existing detectors [16,17,18,19], and focused on associating the detection results with the trajectories across frames in the image sequences [3,20,21]. The simple online and real-time tracking (SORT) associated the detection results by computing the distance between the locations of the currently detected boxes and the predicted boxes via Hungarian algorithm [20]. Predicted boxes were estimated with Kalman Filter (KF) [22]. Only associating the data with positional information was insufficient, which caused unstable trajectories [23]. DeepSORT further augmented a metric based on the similarity of features in the data association method [21]. Following the two works, efforts have been devoted into exploring more discriminative metrics on features and positional information [23,24,25,26,27]. For obtaining better predicted boxes, some methods merged the detection scores into the KF [23,24] or adopted the camera motion compensation (CMC) [25,26,27]. For better association based on the features, additional neural networks for feature extraction was applied to obtain the similarity metrics for the features [2,28,29]. In general, a large number of TBD methods have been proposed, and leading performance has been achieved. Nevertheless, the high performance of the TBD methods relied on strong detectors and complex models, which restricted their applications.

### 2.2. Joint-Detection-and-Tracking

The joint-detection-and-tracking (JDT) methods aimed to detect and track multiple objects in one stage. The pioneering works conducted the object detection in current frame, and performed the data association on two successive frames [30,31]. The CenterTrack then took the objects as points, and performed a tracking-conditional detector to detect objects and associate the data [32]. JDE [2] and FairMOT [33] built object detection networks and ReID network with shared feature extraction networks. Moreover, as Transformer [34] has been introduced in computer vision and impressive performance was achieved, it has been applied in MOT [7,8]. TransTrack built the MOT network with a detection network to obtain the detection boxes with object queries, and augmented an additional decoder for estimating the predicted boxes based on track queries [7]. By computing the IoU of two sets of boxes, it associated the detection boxes and predicted boxes, which was corresponding to the trajectories. TrackFormer directly applied the track queries from the previous frame as the inputs of the decoder in the current frame, and detected the objects with both locations and identities [8].

### 2.3. Transformer-Based MOT Method

Transformer has been a popular architecture in computer vision [34], and significant performance improvements have been achieved in numerous tasks, i.e., classification [35,36], detection [37,38], and segmentation [39]. The Vision Transformer (ViT) introduced a pure transformer network for computer vision tasks, which flatten the images into tokens and process them with stacked layers of multi-head self-attention [40]. The performance improvements were gained for the global context modeling ability of the transformer. However, the local information was neglected and the computational cost was high. Some works proposed designs into ViT to learn local context information [36,41]. Others devoted to reduce the computation and memory cost of the vanilla multi-head self-attention [42,43,44]. For the detection task, DETR [37] built the detection network with encoder–decoder architecture based on transformer, and utilized learnable queries to predict objects without non-maximum suppression. Many follow-up studies have explored the methods to address the slow convergence of DETR by reforming the learnable queries [10,45,46].

## 3. Proposed Methodology

In this section, the mechanism of a representative JDT method, TransTrack, is revisited firstly, where the proposed methods are implemented. The anchor-based queries are then presented for the JDT methods. Following this, the template matching is sketched. Finally, the training and interfering of the new tracker based on the proposed modules are detailed.

### 3.1. Revisit of the TransTrack

The transformer-based TransTrack [7] formulates the object detection and data association in one network, which consists of four main parts, i.e., backbone, encoder, decoder, and prediction heads. The backbone extracted the features of images. The ResNet-50 [47] and FPN [16] are implemented as the backbone. The input of the encoder is the feature map of the image, and the encoder learned the long-range dependency information across the tokens in the feature map. It stacks multi-head self-attention layers and feed-forward networks (FFN) in one block. For the decoder, two parallel modules were applied to detect objects based on the object queries for the current frame and the track queries from the previous frame, respectively, [7]. The two modules have the same architecture, which is built with multi-head self-attention layers, multi-head cross-attention layers and FFN. The inputs for the two modules are the output of the encoder and learnable queries. The difference of the two modules is the query types in the inputs, i.e., the object queries and the track queries. The object queries are a set of learnable parameters, and trained with the other parameters for object detection. The track queries are the reserved output of the module used for detection from previous frame, which are the features of the detected objects in the previous frame. With object queries and track queries, the detection boxes and the track boxes are predicted with two prediction heads, which are the feed forward networks. The data association is conducted by calculating the box IoU scores of the predicted boxes sets. And the Hungarian algorithm is applied for associating each track box with the detection box based on the IoU scores. For the unmatched detection boxes, new trajectories are created. The trajectories of the unmatched track boxes are kept a fixed number of frames since the objects in the trajectories may blocked or disappeared. Although TransTrack accomplish the detection and data association in one network, the slow training convergence and insufficient data association limit its applications.

### 3.2. Anchor-Based Query

The slow training convergence of the TransTrack is caused by inappropriate design of the learnable queries. The queries are fed into the cross-attention module to learn an attention map with keys, and the content information and spatial information are contained in the queries and keys. It can be described as follows.

(1) $${\left(C_{q}+S_{q}\right)}^{T}\left(C_{k}+S_{k}\right)=C_{q}^{T}C_{k}+C_{q}^{T}S_{k}+S_{q}^{T}C_{k}+S_{q}^{T}S_{k}$$

where $C_{q}$ and $C_{k}$ are the content part in the queries, $S_{q}$ and $S_{k}$ are the spatial part in the keys. The content part of the queries not only need to match the content information in the keys, but also need to match the spatial information in the keys [10]. Moreover, the spatial information in the queries randomly initialized, while the spatial information for the keys are generated with the sinusoidal function function [9]. Therefore, the anchor-based-query is proposed to augment explicit prior spatial knowledge into the queries, which encodes the spatial information of the queries with the sinusoidal function function, to accelerate the training.

**Figure 2:**
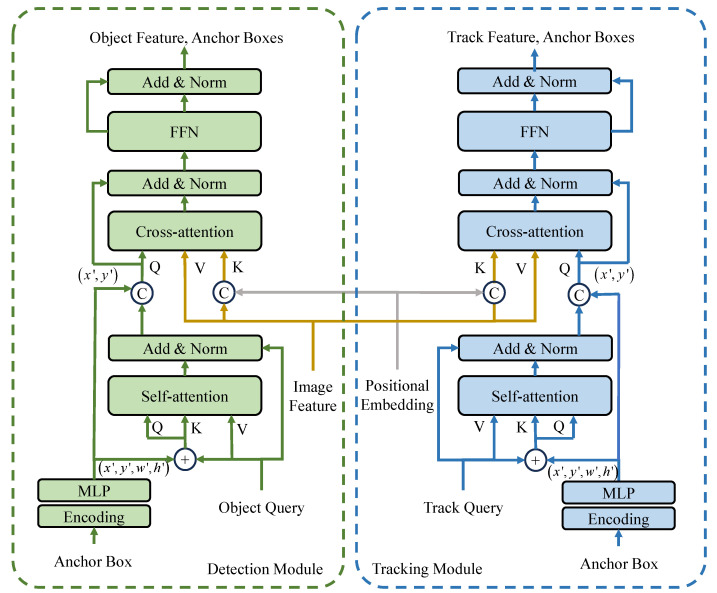
Illustration of the architecture of the decoder based on Anchor-based Query.

The anchor-based-query is implemented in the decoder of the tracking network. As shown in Figure 2, two parts are included in the anchor-based-query (ABQ), i.e., content query and spatial query. Specifically, the content queries and spatial queries for the detection module are the object queries and the anchor boxes, and that for the track module are the track queries and the anchor boxes. The two sets of anchor boxes are generated independently. The content query is same to that in TransTrack, while the spatial query formulates the spatial information in 4D anchors, i.e., $\left(x,y,w,h\right)$, which includes both the position and size of the anchor box. The queries are utilized to probe the features of the images, which leads to directly prediction without non-maximum suppression. The 4D anchor boxes are concatenated with the content queries in the channel domain to match the content information and spatial information separately. Therefore, the locations, $\left(x,y\right)$ in the 4D anchor boxes, are generated to math the positional embeddings in the keys, which include the locations $\left(x,y\right)$. The sizes, $\left(w,h\right)$, are utilized for learning the self-attention of the queries with size information, which can adjust the attention maps with scale information. Each element in the anchor boxes is a learnable parameter, which can be learned in the training procedure. As shown in Figure 2, similar ABQ methods are applied in the two modules of the decoder, the detection module and the track module.

For the detection module, self-attention layers and cross-attention layers are used for query updating and feature investigation. The anchor box is defined as $B=\left(x,y,w,h\right)$. The positional encoding for each element in the anchor can be calculated as follows.

(2) $$\left\{\begin{array}{c} x_{2i}^{\prime }=\sin\left(\frac{x}{T_{2i}/D}\right)\\ x_{2i+1}^{\prime }=\cos\left(\frac{x}{T_{2i+1}/D}\right) \end{array}\right.$$

where *T* is a hand-craft temperature as it in [9], 2i and 2i+1 are the indices, *D* is the half of the dimension of the content queries. Specifically, the dimension of the content queries is 256 in this work. The positional encoding generates a vector with 128 channels from a float, i.e., the positions. For the self-attention learning, the ABQ can be obtained as follows.

(3) $$B_{q}=MLP\left(CAT\left(x^{\prime },y^{\prime },w^{\prime },h^{\prime }\right)\right)$$

where, $MLP\left(•\right)$ denotes the multiple layer perceptron, which consists of 2 layers of linear layer with ReLU activation. $CAT\left(•\right)$ stands for concatenation operation. The output of the concatenating operation has 2×D channels, and the MLP operation reduce the dimension of it to *D* channels so that the spatial queries can be concatenated with the content queries. The queries and keys for the self-attention are the combination of the spatial queries and the content queries as follows.

(4) $$\left\{\begin{array}{c} Q_{s}=C_{q}+B_{q}\\ K_{s}=C_{q}+B_{q} \end{array}\right.$$

where $Q_{s}$ and $K_{s}$ represent the queries and keys for the self-attention learning, respectively, $C_{q}$ is the content item, which is a set of learnable parameters similar to the object query in TransTrack. The values equal the content item. For the cross-attention, the keys contain a feature map and the corresponding positional embedding, the feature map is outputted from the encoder. The values are the image features same to the keys. They are obtained as follows.

(5) $$\left\{\begin{array}{c} K_{c}=CAT\left(F,P\right)\\ V_{c}=F \end{array}\right.$$

where $K_{c}$ and $V_{c}$ represent the keys and values for the cross-attention, respectively, *F* and *P* stand for the feature map and the corresponding positional embedding. The queries for the cross-attention learning is the union, which consists of content query and the locations in the spatial query, which can be described as follows.

(6) $$Q_{c}=CAT\left(C_{q}^{\prime },CAT\left(x^{\prime },y^{\prime }\right)•MLP_{c}\left(C_{q}\right)\right)$$

where $C_{q}^{\prime }$ represents the content queries outputted from the self-attention layer, $C_{q}$ is the content queries same to that in Equation (4), $x^{\prime }$ and $y^{\prime }$ are the locations. $MLP_{c}\left(•\right)$ is a multiple layer perceptron, which is utilized to learn a scale reference map on the content query, and • is the element-wise multiplication. The concatenations of the content information and spatial information in the queries and keys enable the cross-attention learning, and decouple the matching of the content information and spatial information.

The content queries and spatial queries for the detection module are sets of learnable parameters, and the queries for the tracking module are the outputs of the detection module. Specifically, the track queries and the ABQ for the tracking module are the object features and anchor boxes outputted from the detection module in the previous frame. The anchor boxes are the positional encoding corresponding to the ABQ.

### 3.3. Template Matching

To enable data association in JDT methods with feature information, template matching (TM) is augmented in the network. The TBD methods applied additional networks to match the features of the detected objects and the objects in the trajectories. This solution is not suitable for the JDT methods due to the additional network increase the complexity of the JDT methods. Meanwhile, these features are already been extracted in the networks of JDT methods. Therefore, the TM utilizes the features from the tracking networks to compute the feature similarity scores. By comparing the features of the detected objects and the templates corresponding to the trajectories, the ABQ-Track can associate the detection results and trajectories with additional feature information. Moreover, the tracker can maintain the trajectories with long-time memory by integrating the former features into the corresponding templates.

Considering the $F_{Track}^{t}$ is the feature map outputted from the right branch in the decoder as shown in Figure 2, the superscript *t* and subscript Track represent the number of the frame and the identity of the trajectory. The template for each trajectory can be computed as follows.

(7) $$T_{Track}=\sum_{t=M-m}^{M}w_{Track}^{t}F_{Track}^{t}$$

where $T_{Track}$ represents the template corresponding to the trajectory, Track, $w_{Track}^{t}$ denotes the learnable weights of the features, *M* and *m* are the number of current frame and the total number of features, respectively. Taking $F_{Object}$ as the features of detected object, which is the output of the left branch in decoder as shown in Figure 2, each element in the feature similarity score map is obtained with mahalanobis distance as follows.

(8) $$S_{Feature}^{i,Track}=MD\left(F_{Object}^{i},T_{Track}\right)$$

where *i* and Track represents the index of detected objects and the templates, respectively. The $MD\left(•\right)$ represents the mahalanobis distance function.

According to Equation (8), the more similar the two features are, the lower $S_{Feature}^{i,Track}$ will be. However, the score map based on spatial information is computed with IoU, which is higher when two boxes share larger overlapping area. Hence, the feature similarity score is further processed as follows.

(9) $$S_{Feature}^{i,Track}=1-Norm\left(S_{Feature}^{i,Track}\right)$$

where $Norm\left(•\right)$ is the normalization operation.

### 3.4. Architecture

**Figure 3:**
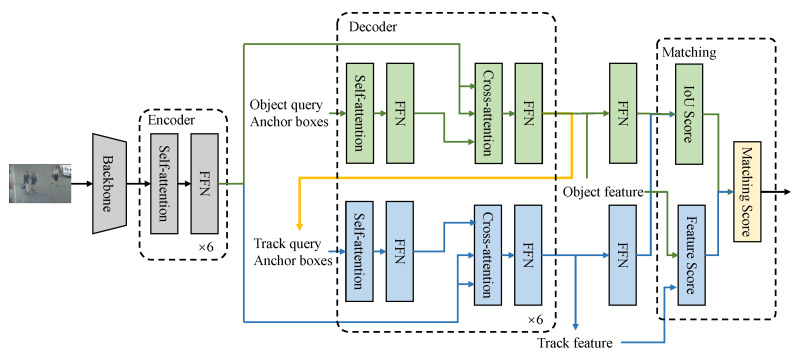
Illustration of the pipeline of the proposed tracker, ABQ-Track.

The pipeline of the ABQ-Track is illustrated in Figure 3. The two methods proposed in this paper is applied in the decoder and the matching section of the network. For fair evaluation, the rest parts of the network share the same structure to the TransTrack. In the decoder, 6 blocks are implemented for each module, which is described in Section 3.2. The number of the content queries is set to 500, which is based on the experimental results. And the dimension of the content queries is set to 256. The spatial queries for the self-attention and the cross-attention in the decoder have the dimension of 256, and the number is 500. The keys for the self-attention shares the same size with the queries. The keys for the cross-attention are obtained by concatenating the feature outputted from the encoder and the positional embedding. The decoder outputs two sets of feature maps and anchor boxes corresponding to the object queries and the track queries. The feature maps are fed into the two prediction heads to obtain two prediction maps, $m\in R_{500\times 4}$, which indicates the locations of the objects detected with the detection module and the track module. The IoU scores are computed with the locations of the two set of objects. The feature maps are also fed into the TM module to obtain the feature scores.

### 3.5. Training and Inference

The tracking network is trained with data from CrowdHuman [48] and MOT [13,14]. The data are randomly sampled from a real video clip in the two data sets. Due to the two modules in the decoder perform predictions in the same images, the two modules can be trained with same loss function. Following [37], the loss function of the tracking network can be formulated as follows.

(10) $$L=\lambda_{1}L_{cls}+\lambda_{2}L_{L_{1}}+\lambda_{3}L_{giou}$$

where $\lambda_{1}$, $\lambda_{2}$ and $\lambda_{3}$ stand for the weights of the losses. $L_{cls}$ represents the focal loss of classification [17]. $L_{L_{1}}$ and $L_{giou}$ are the L1 loss and generalized IoU loss [49] for the regression of the coordinates of the predicted boxes.

In the initial image of a given sequence, the ABQ-Track detects only the objects with the parts for detection in the network. Then, the detection and data association are conducted on the rest images of the sequence with the whole tracking network. Two sets of predicted box can be obtained with the features outputted from the decoder based on the object query and track query, respectively. The IoU similarity score map between the two sets of box is computed with the Kuhn-Munkres algorithm. Additionally, the template matching module outputs the similarity score map based on the features of the detected objects and the object within each trajectory. The finally score map can be obtained with the two score maps with weights as follows.

(11) $$S=w_{IoU}S_{IoU}+w_{Feature}S_{Feature}$$

where $S_{IoU}$ and $S_{Feature}$ stand for the score maps of the IoU and feature similarity, $w_{IoU}$ and $w_{Feature}$ represent the corresponding learnable weights. Based on the score map, the traditional Hungarian algorithm [20] is applied for data association. For the unmatched boxes based on the object query, new trajectories are birthed for them. And for the trajectories who have not associated to any boxes based on the object query for 30 consecutive frames, the trajectories are removed. In this way, each box based on the object query has a unique ID.

## 4. Experiment

To evaluate the proposed methods, experiments have been conducted on commonly applied data sets for multiple object tracking, MOT17 [13] and MOT20 [14]. An ablation study on the effectiveness of proposed methods is presented, and the comparison of the proposed tracker and the other MOT trackers are given to show that the proposed tracker has competitive performance.

### 4.1. Implementation Details

The network of the ABQ-Track is trained on 2 datasets, CrowdHuman and MOT. The CrowdHuman is utilized to train the network firstly, which is a dataset on scenarios of dense pedestrians. 15,000 training images and 470,000 instances are contained in the CrowdHuman, which provides more categories of the samples. Then, the network is trained with CrowdHuman and MOT. The training data in MOT is split into two subsets for training and validation, respectively. Regular data augmentations are applied on the training data, which include random horizontal, random crop and scale augmentation. The trained tracking network is tested locally with the testing data in MOT dataset, and the results are uploaded to the given server for evaluation. The evaluation metrics in the experiments are MOTA, IDF1, FP, FN, IDS, et al., which follow that in [13]. MOTA is the metric specially designed for MOT task, which is calculated with false positive (FP), false negative (FN), ID switches (IDS) and ground truth (GT).

The backbone in the proposed ABQ-Track is ResNet-50, which is same with the TransTrack. The encoder and decoder have 6 blocks. In each block of the encoder, a self-attention module and a FFN is applied. The structure of the decoder blocks is shown in Figure 2. Each of the two prediction heads have 3 layers of perceptron with ReLU activation function and a linear projection layer. The parameters of the backbone are initialized with that learned on ImageNet [50], and the rest parameters are initialized with Xaxiver-init [51]. There are two steps of training of the tracking network, and the optimizer is AdamW. Firstly, the tracking network is trained on the CrowdHuman for 50 epochs, and the batch size is set to 16. The learning rate is set to $2\times 10^{-4}$, and it drops to $2\times 10^{-5}$ after 30th epoch. Secondly, the tracking network is fine-tuned for 40 epochs, including CrowdHuman and the splits of MOT. The learning rate and the batch size for the fine-tuning are set to $2\times 10^{-5}$ and 16. Commonly used data augmentations, i.e., random horizontal, random crop, scale augmentation, are applied for the two training steps. The weights of the losses are set to $\lambda_{1}=2$; $\lambda_{2}=5$; $\lambda_{3}=2$.

### 4.2. MOT Challenge Test Results and Discussion

#### 4.2.1. MOT17

The upper section of Table 1 reports the evaluation results of ABQ-Track and 10 other trackers on the MOT17 test dataset. ABQ-Track, which is pre-trained with the CrowdHuman dataset, achieved the best score in MOTA, FP and FN. In terms of the other metrics, competitive performance is achieved. The better performances of SOTMOT, CSTrack and TransCenter in IDF1 are achieved with larger pre-train data set. ABQ-Track chooses the CrowdHuman for fair comparison with TransTrack. Comparing to TransTrack, ABQ-Track achieves better performance in MOTA, IDF1, FP, MT, ML and IDS. The better scores in MT and ML can be attributed to the implementation of the ABQ, which improves the detection performance of the tracking network. The utilization of TM reduces the IDS, for the feature mathcing stablizing the trajectories. The ABQ and TM work together to improve the performance in MOTA. Notably, the network of TransTrack is needed training for 150 epochs, while that of ABQ-Track is trained in only 50 epochs.

#### 4.2.2. MOT20

The lower section of Table 1 presents the performance of ABQ-Track and the other 5 trackers on the MOT20 test dataset. MOT20 consists of 4 training sequences and 4 testing sequences, which are obtained in more challenging tracking scenarios. Specifically, the test data in MOT20 contain extremely dense scenarios. As shown in Table 1, ABQ-Track achieves competitive performance in the comparison with the other 5 trackers. The trackers, i.e., CSTrack, CorrTrack, SOTMOT, surpass the performance of ABQ-Track for larger training dataset. However, the IDS of ABQ-Track is lower for the utilization of the TM. Compared to TransTrack, ABQ-Track achieves better performance in all the metrics. which reveals that the proposed modules improves the performance of the JDT trackers in dense multiple object tracking scenaros.

### 4.3. Ablation Study

#### 4.3.1. Anchor-Based-Query

In this section, the ABQ-Track networks trained for different numbers of epochs are evaluated on the validation data in MOT17 since that the motivation of utilizing the ABQ is to reduce the training epochs of JDT methods. Table 2 reports the performance results of the trackers in this experiment. All models are trained with the same data. TransTrack, ABQ-10, ABQ-25, and ABQ-50 are trained in 150 epochs, 10 epochs, 25 epochs and 50 epochs, respectively. The tracker with ABQ achieves convergence in 50 epochs, and further training brings no performance improvement. Comparing to TransTrack, the tracker with ABQ has faster training procedure and better performance. As the queries in the decoder works as pooling feature from a feature map. The content queries represents the semantic information, while the spatial queries constrain the pooling feature around the content queries. The Anchor-based-Query leads the tracker to focus on the local area around the corresponding object. This is the main reason for the better performance and faster convergence than that of TransTrack.

Moreover, the performances of the trackers with different ABQ designs are reported in Table 3. The Location represents the generating ABQ locations. The Adding stands for the augmenting the ABQ into the object queries and track queries with summation. The ABQ in the table represents the methods described in Section 3.1. All trackers are trained for 50 epochs, and with the same training data. The None tracker achieves lowest performance scores since it needs more epochs to convergence. The Adding tracker have similar performance as the None tracker due to summation mixes the content queries and anchor-box queries for the attention learning. Comparing the Location and ABQ, adding scale information in the ABQ improves the performance of the trackers.

Additionally, the comparison on performances of the trackers with different numbers of the ABQ is conducted due to the quantity of queries has obvious affect on the performance of the transformer-based detection methods. The experimental results are reported in Table 4. Comparing the trackers with 300 anchor-based-queries and 500 anchor-based-queries, more anchor-based-queries improve the performance of the tracker. And further adding more anchor-based queries does not lead to obvious performance improvement. This is because too many anchor-based queries leads to excessive fragmentation of the feature map, which results in confusion between the localization targets in dense scenarios. Based on this experiment, the number of the anchor-based-queries in ABQ-Track is set to 500.

#### 4.3.2. Template Matching

The classical JDT methods, TransTrack, focus on associating the detected objects and trajectories based on the spatial score, i.e., IoU score. Id switches often occur when objects are occluded during tracking or objects with similar appearances appear. Preserving historical feature information can benefit for maintaining the stable trajectories. Hence, to avoid identity switches and maintain stable trajectories, the TM module is designed for TransTrack. The TM module bring historical feature information, add feature-based score into the data association method in the tracker. In the section, the performance comparison is made between the trackers with or without the TM module. Meanwhile, the amount of past feature information saved in the template is evaluated. The results are reported in Table 5.

In Table 5, the None represents the ABQ-Track without the TM module. TM-Input stands for the ABQ-Track with the TM module, and only one feature is saved to obtain the template. TM-5, TM-10, TM-20, TM-30 stand for the TM utilizes 5, 10, 20 and 30 features from the past to obtain the template, respectively. As shown in Table 5, trackers apply the TM modules outperform the tracker without the TM modules, which verifies the effectiveness of the TM module. Moreover, TM-30 achieves the best performance among the trackers with the TM module. Hence, the ABQ-track in Table 1 utilizes 30 features from the past to obtain the template.

#### 4.3.3. Visualization of Experimental Results

**Figure 4:**
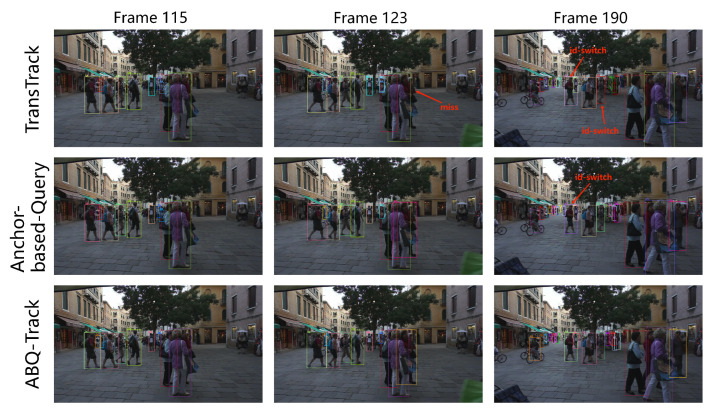
Visualization of tracking results of TransTrack, tracker with ABQ and ABQ-Track.

To better illustrate the enhancement in MOT tracking performance with the ABQ and TM, the visualization of the tracking results of the trackers in one sequence is presented in Figure 4. These results are all derived from the MOT17-02 validation video sequence. The first row represents the tracking results of TransTrack, the second row depicts the results of the tracker applying the ABQ, and the third row illustrates the tracking result of the ABQ-Track.

As shown in Figure 4, the tracker with Anchor-based query detected the object which is missed by the TransTrack, and certain ID switch in the results of TransTrack does not occur in the results of the tracker with Anchor-based query. This reveals that the ABQ improves the detection performance and reduces the ID switches of the tracker. Comparing the second and third row, the ID switches is further reduced, which verifies the effectiveness of the TM.

## 5. Conclusions

In this paper, a JDT method based on a transformer, ABQ-Track, is proposed. First, the explicit spatial knowledge is augmented in the query of the decoder with a four-dimensional anchor box (ABQ). The ABQ increases the performance of the tracker, and leads to faster training convergence. Additionally, a new template matching module (TM) is proposed to enable the JDT methods to associate the trajectories and detection results based on historical features. Based on the two modules, the ABQ-Track is built by modifying the classical JDT method, TransTrack, and achieves better performance than TransTrack on MOT 17 and MOT 20, 75.9% and 66.3% in MOTA, respectively. Specifically, the ABQ-Track achieves the better performance after being trained for 50 epochs, while the TransTrack needs to be trained for 150 epochs.

## Figures and Tables

**Table 1:** Results on the MOT17 and MOT20 Test Sets: The upper section presents results on private detection in the MOT17 dataset, while the lower section displays the detection outcomes on the MOT20 dataset.The upward (downward) arrows in the table indicate that the larger (smaller) the parameter, the better the corresponding performance.

Method	MOTA↑	IDF1↑	FP↓	FN↓	MT↑	ML↓	IDS↓
MOT17							
UMA [52]	53.1	54.4	22,893	239,534	21.5	31.8	2251
TubeTK [53]	63.0	58.6	27,060	177,483	31.2	19.9	4137
CenterTrack [32]	67.8	64.7	18,489	160,332	34.6	24.6	3039
QuasiDense [2]	68.7	66.3	26,589	146,643	43.8	17.22	3378
TraDeS [54]	69.1	63.9	20,892	150,060	36.4	21.5	3555
SOTMOT [55]	71.0	71.9	39,537	118,983	42.7	15.3	5184
TransCenter [56]	72.5	58.1	25,722	114,310	64.7	12.2	2332
FairMOT [57]	73.7	72.3	27,507	117,477	43.2	17.3	3303
TransTrack [7]	74.5	63.9	28,323	112,137	46.8	11.3	3663
CSTrack [58]	74.9	72.6	23,847	114,303	41.5	17.5	3567
ABQ-Track (ours)	75.9	65.4	16,977	115,667	52.8	2.8	3135
MOT20							
FairMOT [57]	61.8	67.3	103,440	88,901	66.3	8.5	5243
TransTrack [7]	64.5	59.2	28,566	151,377	49.1	13.6	3565
CorrTracker [59]	65.2	69.1	79,429	95,855	66.4	8.9	5183
CSTrack [58]	66.6	68.6	25,404	144,358	50.4	15.5	3196
ABQ-Track (ours)	66.3	60.3	20,179	149,536	38.0	45.1	3383

**Table 2:** Comparison of the tracker with ABQ trained in different epochs. The experiment is conducted on the validation data in MOT17. The IDs represents the percentage of the number of the ID switches to all the identities. The upward (downward) arrows in the table indicate that the larger (smaller) the parameter, the better the corresponding performance.

Architecture	MOTA↑	FP↓	FN↓	IDF1↑	IDs↓
TransTrack	65.0%	4.3%	30.3%	-	0.4%
ABQ-10	37.8%	33.1%	68.5%	44.8%	17.1%
ABQ-25	54.8%	19.3%	45.4%	56.8%	13.7%
ABQ-50	66.1%	3.9%	28.6%	66.7%	0.6%
ABQ-75	65.8%	4.1%	29.0%	60.2%	10.5%

**Table 3:** Comparison of the trackers with different ABQ designs. The upward (downward) arrows in the table indicate that the larger (smaller) the parameter, the better the corresponding performance.

Trackers	MOTA↑	FP↓	FN↓	IDF1↑	IDs↓
None	54.5%	16.0%	33.7%	57.9%	13.9%
Location	64.8%	4.3%	29.6%	65.2%	0.6%
Adding	55.3%	15.8%	33.8%	58.2%	13.1%
ABQ	66.1%	3.9%	28.6%	66.7%	0.6%

**Table 4:** The experimental results on the comparison of different anchor-based-queries settings. The upward (downward) arrows in the table indicate that the larger (smaller) the parameter, the better the corresponding performance.

num_Query	MOTA↑	FP↓	FN↓	IDF1↑	IDs↓
300	66.7%	6.6%	25.3%	63.7%	1.4%
500	68.6%	4.3%	26.2%	70.1%	0.9%
900	68.4%	5.5%	24.9%	68.3%	1.1%

**Table 5:** Performance comparison for TM modules. The upward (downward) arrows in the table indicate that the larger (smaller) the parameter, the better the corresponding performance.

Trackers	MOTA↑	FP↓	FN↓	IDF1↑	IDs↓
MOT-None	66.1%	3.9%	28.6%	66.7%	0.6%
TM-Input	66.4%	5.5%	25.5%	67.1%	1.2%
TM-5	66.6%	5.5%	25.5%	67.7%	1.3%
TM-10	66.9%	5.4%	25.1%	67.9%	1.2%
TM-20	67.6%	4.7%	24.9%	68.9%	1.3%
TM-30	68.6%	4.3%	26.2%	70.1%	0.9%

## Data Availability

The public data used in this work are listed here: CrowdHuman www.crowdhuman.org/ (accessed on 28 November 2023), MOT17 https://motchallenge.net/ (accessed on 28 November 2023), MOT20 https://motchallenge.net/ (accessed on 28 November 2023).
